# Molecular detection of Shiga toxin and extended-spectrum beta-lactamase (ESBL)-producing *Escherichia coli* isolates from sheep and goats

**DOI:** 10.1007/s11033-023-08987-0

**Published:** 2024-01-02

**Authors:** Tsepo Ramatla, Mpho Tutubala, Tshepang Motlhaping, Lara de Wet, Prudent Mokgokong, Oriel Thekisoe, Kgaugelo Lekota

**Affiliations:** Unit for Environmental Sciences and Management, North West University, Potchefstroom, 2531 South Africa

**Keywords:** *E. coli*, Antimicrobial resistance, Extended-spectrum *β*-lactamases, Virulence factors, Shiga-toxins

## Abstract

**Background:**

The Shiga toxin (Stx)-producing *Escherichia coli* (STEC) have become important global public health concerns. This study investigated the prevalence, antimicrobial resistance profile, and extended-spectrum beta-lactamase-producing *E. coli* in sheep and goat faeces.

**Methods and results:**

A total of 53 *E. coli* isolates were confirmed by PCR targeting the *uidA* [*β*-D glucuronidase] gene. The Shiga toxin genes *stx*1 and *stx*2, as well as *bfp*A, *vir*, *eae*A, *lt* and *aaf*II virulence genes, were detected in this study. Of the 53 isolates confirmed to be STEC, 100% were positive for *stx2* and 47.2% for *stx1*. Three isolates possessed a combination of *stx*1 + *stx*2 + *eae*A, while four isolates harboured *stx*1 + *stx*2 + *vir* virulence genes. The isolates displayed phenotypic antimicrobial resistance against erythromycin (66.04%), colistin sulphate (43.4%), chloramphenicol (9.4%) and ciprofloxacin (1.9%). A total of 28.8% of the strains were phenotypically considered ESBL producers and contained the beta-lactamase *bla*_*CTX-M-9*_ and *bla*_*CTX-M-25*_ gene groups. A larger proportion of the *E. coli* strains (86.8%) contained the antibiotic sulphonamide resistant (*sul*II) gene, while 62.3%, 62.3%, 52.8%, 43.4%, 41.5%, 20.8%, 18.9%, 11.3%, 11.3%, 9.4%, 9.4% and 5.7% possessed *mcr-4, floR, mcr-1, tet*(A), *sulI, tet*(O), *tet*(W), *parC, mcr-2, ampC 5, qnrS* and *ermB* genes, respectively. Thirteen isolates of the ESBL-producing *E. coli* were considered multi-drug resistant (MDR). One Shiga toxin (*stx2*) and two beta-lactamase genes (*bla*_*CTX-M-9*_ and *bla*_*CTX-M-25*_ groups) were present in 16 isolates. In conclusion, the *E. coli* isolates from the small stock in this study contained a large array of high antibiotic resistance and virulence profiles.

**Conclusions:**

Our findings highlight the importance of sheep and goats as sources of virulence genes and MDR *E. coli*. From a public health and veterinary medicine perspective, the characterization of ESBL producers originating from small livestock (sheep and goats) is crucial due to their close contact with humans.

**Supplementary Information:**

The online version contains supplementary material available at 10.1007/s11033-023-08987-0.

## Introduction

*Escherichia coli* is a rod-shaped, Gram-negative coliform bacteria that is widely distributed in nature. They are a large and diverse group of bacteria that infects both humans and animals [1]. The Shiga toxin (*Stx*)-producing *Escherichia coli* (STEC) is an important zoonotic foodborne pathogen causing gastrointestinal complications in humans [2]. The term STEC refers to a pathotype of *E. coli* that produces *Stx1* and/or *Stx2* [2, 3]. There are two major families of Shiga toxins (*Stx*), *Stx1* and *Stx2*, with 70 percent similar amino acid sequences [2, 4]. It is less likely that *Stx1* will produce diseases in humans, while *Stx2* is more likely to cause diseases like hemorrhagic colitis (HC) and hemolytic-uremic syndrome (HUS) [5].

More than 200 serotypes of STEC have been identified [6]. Due to its ubiquitous nature and sharing the same niche as other enteric pathogens and being transmissible by the same route, it contributes to the dissemination of antimicrobial resistance (AMR) [7]. Globally, AMR is considered a major threat to public health [7, 8] and affects both clinical settings and the community at large [9]. In the sentinel organism *E. coli*, resistance proportions are often called “prevalence” [10]. Prevalence is used to measure the proportion of bacteria that are resistant to a particular antibiotic. This can be used to predict the spread of antibiotic resistance in a population. Prevalence is an important factor in understanding the impact of antibiotics on bacterial populations, particularly as farmers use antibiotics as animal fodder [11].

Antibiotics such as *β*-lactams can cause multidrug resistance in *E. coli* isolates, mostly due to the synthesis of extended-spectrum *β*-Lactamases (ESBL) and/or plasmid-mediated *amp*C *β*-lactamases [12, 13]. The genes that encode ESBL enzymes are found on plasmids or on single chromosomes of bacteria, which also contain genes for resistance to other antimicrobial agents, including quinolones, aminoglycosides, trimethoprim, sulphonamides, tetracyclines and chloramphenicol [14]. As plasmids can be transferred between bacterial species, the spread of ESBL enzymes is a major concern, as they are often accompanied by other genes that impart resistance to other antimicrobial agents, making them more difficult to treat.

Beta-lactam resistance is caused by the production of beta-lactamases such as ESBLs, metallo-β-lactamases (MBLs) and sometimes *amp*C plasmid *β*-lactamases [15]. These enzymes confer resistance to a broad range of *β*-lactam antibiotics, including penicillin and cephalosporins. As a result, treatment of infections caused by *β*-lactamase-producing bacteria is difficult. Multiple antibiotic resistance genes are a trait of Gram-negative bacteria, which has effects on treatment and the environment. Gram-negative bacilli such as *Klebsiella pneumoniae*, *Acinetobacter baumannii*, *Pseudomonas aeruginosa*, *E. coli* and *Enterobacter* spp. all possess the ESBL genes *bla*_*CTXM-1*_, *bla*_*TEM*_ and *bla*_*CTXM-*8_ as well as the carbapenemase genes *bla*_*NDM*_, *bla*_*IMP*_, *bla*_*OXA*_ and *bla*_*VIM*_ [16, 17]. From the “One Health” perspective, the World Health Organization (WHO) has identified ESBL-producing *E. coli* as an indicator of antimicrobial resistance [9, 18]. This means that monitoring the spread of ESBL-producing *E. coli* can provide a snapshot of the prevalence of antimicrobial resistance in a given area. It also helps inform public health interventions that can help reduce the spread of antimicrobial resistance.

Due to the widespread use of antibiotics, ESBL-producing *E. coli* with virulence and resistance profiles have been identified from livestock more frequently, which has facilitated the establishment of infections that are multidrug resistant. There are not many reports of *E. coli* isolates causing AMR in sheep and goats in South Africa. The current study therefore sought to ascertain the frequency of *E. coli* generating extended-spectrum beta-lactamases (ESBL) and the Shiga toxin in sheep and goats in the Matlwang community in Potchefstroom, North-West Province, South Africa.

## Materials and methods

### Ethical statement

The study was approved by the Faculty of Natural and Agricultural Sciences Ethics Committee (NWU-00776-21-A9) of North‒West University.

### Study area and sample collection

The sample collection in this study was carried out in the small farming community of Matlwang in Potchefstroom, North West Province, South Africa. Samples were collected between February 2022 and April 2022. Fecal samples were collected from four different sheep and goat flocks, which were selected randomly. A total of 57 fecal samples (37 from sheep and 20 from goats) were collected from the study site and were isolated for the presence of *E. coli*. Immediately after collection, samples were packaged in clear polyethylene bags, kept in a cooler box and transported to the laboratory. Samples were processed within three to five hours after collection.

### Microbiological techniques and analysis

Five grams of each animal fecal sample was added to 45 ml of peptone enrichment broth for Enterobacteriaceae and incubated at 37 °C for 24 h. The selective media used was sorbitol MacConkey agar (SMA). The enriched broth was inoculated on SMA using the spread plate method. Bacterial growth was enumerated and identified after 24 h of incubation at 37 °C on SMA. The *E. coli* appears yellowish on MacConkey agar because it does not ferment sorbitol or ferments it at a very slow rate. The isolates were confirmed as presumptive pathogenic *E. coli* isolates, after phenotypic identification. Pure isolates were preserved in 20% glycerol (Merck, SA) and stored at − 80 °C for further analysis.

### Bacterial genomic DNA extraction

Genomic DNA (gDNA) extraction was performed on the 55 presumptive *E. coli* isolates. Bacterial cells grown overnight were used to extract genomic DNA. A chromogenic DNA kit (ZYMO Research Quick-DNA Microbe Mini-prep commercial Kit) was used to extract and purify the genomic DNA by following the manufacturer’s protocol. A NanoDrop Lite 1000 spectrophotometer (Thermo-Fisher Scientific, USA) was used to determine the concentration and purity of DNA, which was ultimately stored at − 80 °C until further analysis.

### Identification of diarrheagenic *Escherichia coli* using *uidA* PCR assay

A PCR assay was conducted to amplify the *E. coli* housekeeping *uidA* [*β*-D glucuronidase] gene as described by Omolajaiye et al. [19] using the following primers: UidA-F AAAACGGCAAGAAAAAGCAG and UidA-R ACGCGTGGTTACAGTCTTGCG, which produces a 147 bp amplicon size. The PCR consisted of 12.5 μL of AmpliTaq Gold® DNA Polymerase, 0.05 units/L Gold buffer, 930 mM Tris/HCl pH 8.05, 100 mM KCl, 0.4 mM of each dNTP, and 5 mM MgCl2] (New England Biolabs, USA), 8.5 μL of RNase-nuclease free PCR water, 1 μL of 10 μM each primer and 2 μL of template gDNA. The cycling consisted of an initial denaturation at 95 °C for 5 min, followed by 40 cycles of 95 °C for 40 s, 60 °C for 30 s and 72 °C for 40 s and a final extension of 72 °C for 7 min using the ProFlex PCR System (Applied Biosystems, USA). The negative control was a DNA-free template (nuclease-free water). To allow standardization, molecular weight markers of 1 kb and 100 bp DNA (PROMEGA, Madison, WI, USA) were used to determine the size of the PCR amplicons. For product size confirmation and yield estimation, 5 µL of the PCR products were loaded onto 1% agarose gels, subjected to electrophoresis for 45 min at 80 V and stained with ethidium bromide.

### Identification of diarrheagenic *E. coli* using the 16S rRNA gene

The *E. coli* that was positive for the *uidA* PCR assay was further screened by 16S rRNA PCR using the following pairs of primers: 27F: AGA GTT TGA TCM TGG CTC AG and 1492R: GGT TAC CTT GTT ACG ACT T [20]. To set up PCR assays for virulent genes, a total of 25 µL reaction mixture consisted of 12.5 µL of the 2X [AmpliTaq Gold® DNA Polymerase, 0.05 units/L, Gold buffer, 930 mM Tris/HCl pH 8.05, 100 mM KCl, 0.4 mM of each dNTP, and 5 mM MgCl2], 10 µM of each primer, 2 µL of template DNA and 8.5 µL nuclease-free water. PCR conditions were optimized as follows: initial denaturation step at 96 °C for 4 min, followed by 30 cycles of denaturation at 94 °C for 30 s, annealing at 57 °C for 30 s and extension at 72 °C for 1 min and finally a single and final extension step at 72 °C for 10 min. The representative PCR products were cleaned up using ExoSAP-IT (ThermoScientific, USA), subjected to cycle sequencing using the BigDye Terminator v3.1 kit (ThermoScientific, USA), and sequenced on the SeqStudio genetic analyzer at North‒West University, Potchefstroom, South Africa.

### Detection of virulence genes

PCR was used to detect the presence of virulence genes, including the *eaeA* gene of enterohemorrhagic *E. coli* (EHEC), enterotoxigenic *E. coli* (ETEC) *lt*, enteroinvasive *E. coli* (EIEC) *vir*, enteroaggregative *E. coli* (EAEC) *aafII* and Shiga toxin-producing *E. coli* (STEC) (*stx1* and *stx2*). The reaction was carried out in a total volume of 25 μL consisting of 12.5 μL of 2X DreamTaq Green Master Mix (New England Biolabs, USA), 8.5 μL of RNase-nuclease free PCR water, 1 μL of each primer and 2 μL of template DNA. The PCR conditions were performed as described and optimized by Omolajaiye et al. [19] with slight modifications. The confirmation of virulence genes in the isolates are shown in Table 1. The detection of the *stx1* and s*tx2* genes was performed using a multiplex PCR assay [21].

**Table 1:** Primer pairs used in this study to determine the *E. coli* pathotypes of the isolates

Pathotype	Target Gene	Primer	Sequence of primers (5–3′)	Amplification length (bp)	Annealing temp. (°C)	Reference
ETEC	*lt*	FW	GCACACGGAGCTCCTCAGTCTCC	218	56	[19]
		RV	TTCATCCTTTCAATGGCTTT			
EAEC	*aafII*	FW	CACAGGCAACTGAAATAAGTCTGG	378	56	[19]
		RV	ATTCCCATGATGTCAAGCACTTC			
EIEC	*vir*	FW	AGCTCAGGCAATGAAACTTTGAC	822	61	[19]
		RV	TGGGCTTGATATTCCGATAAGTC			
EIEC	*eaeA*	EAE1	TCAATGCAGTTCCGTTATCAGTT	450	55	[19]
		EAE2	GTAAAGTCCGTTACCCCAACCTG			
STEC	*stx1*	EVT1	CAACACTGGATGATCTCAG	349	55	[21]
		EVT2	CCCCCTCAACTGCTAATA			
STEC	*stx2*	EVS1	ATCAGTCGTCACTCACTGGT	110	55	[21]
		EVS2	CTGCTGTCACAGTGACAAA			

### Antimicrobial susceptibility testing and ESBL detection

A Kirby-Bauer disc diffusion technique was used to determine the antimicrobial susceptibility profile of the isolates [22]. The antimicrobial agents selected for this study are some of the most commonly used prophylactics in small and large stocks in South Africa. Aminoglycosides [streptomycin (10 μg)], fluoroquinolones [ciprofloxacin (5 μg)], nalidixic acid (30 μg), macrolides [erythromycin (15 μg)], penicillin beta-lactam [ampicillin (10 μg)], polymyxins [colistin sulfate (300 μg)], and phenicols [chloramphenicol (30 μg)] were obtained from Mast Diagnostics, UK. The pure *E. coli* isolates were inoculated in nutrient broth and incubated at 37 °C for 24 h. The bacterial suspension was spread onto sterile Muller-Hinton agar (MHA) plates using a sterile spreader and allowed to dry for 10 min at room temperature. Then, the antibiotic discs were placed onto the agar plates and incubated at 37 °C for 24 h. After incubation, the diameter of the zone around the colony was measured and compared with the Clinical and Laboratory Standards Institute (CLSI) to categorize it into resistant (R), intermediate (I) and susceptible (S) [23]. A multidrug-resistant isolate was defined as any isolate showing resistance to more than two classes of antibiotics [24]. *Escherichia coli* ATCC 25922 was used as a reference for quality control in the antimicrobial susceptibility test. The ESBL-resistance phenotype was detected using the double disc synergy test (DDST). Using Muller-Hinton agar (Oxoid, UK), pairs of disks containing 30 mg of ceftaxime (CTX) and 30 mg of ceftazidime (CAZ) were inoculated with 20/10 mg of amoxicillin-clavulanic acid (AMC) through the same inoculated plate. A positive test result was defined as a 5 mm increase in the zone diameter compared to that of a disk without clavulanic acid [23]. It was classified as multidrug resistant (MDR) when it was resistant to at least three antimicrobial groups.

### Detection of antibiotic resistance genes by PCR

The gDNA extracted from *E. coli* isolates was screened for the presence of the antibiotic resistance genes tetracycline (*tet*A, *tet*O, *tet*X, *tet*P, *tet*W and *tet*K), erythromycin (*erm*B), colistin (*mcr*-1, *mcr*-2, *mcr*-3, *mcr*-4 and *mcr*-5), chloramphenicol (*cat*I, *cat*II, *cat*III, *cat*IV, and *flo*R), sulfonamide (*sul*I, *sul*II and *sul*III), quinolone (*qnr*A, *qnr*D, *qnr*S and *par*C), and aminoglycoside (*str*A, *str*B and *aad*A). To set up PCR assays for antibiotic resistance genes, a 25 µL reaction mixture consisted of 12.5 µL of the PCR Master Mix (AmpliTaq Gold® DNA Polymerase, 0.05 units/L, Gold buffer, 930 mM Tris/HCl pH 8.05, 100 mM KCl, 0.4 mM of each dNTP, and 5 mM MgCl2), 10 µM of each primer, 2 µL of template DNA, and 8.5 µL nuclease-free water. The amplification program consisted of the following steps: 94 °C for 5 min, followed by 35 cycles of amplification divided into 1 min of denaturation at 94 °C, 1 min of primer annealing at the temperature according to Supplementary Table S1, and 1 min of primer extension at 72 °C, followed by a 10 min final extension step at 72 °C. Furthermore, multiplex PCR screening for *mcr-1* to 5 colistin-encoding genes was performed as described previously [25]. Electrophoresis of PCR amplicons was conducted as described above.

### Molecular screening of β-lactamase-encoding genes

The *E. coli* isolates were screened for the *β*-lactam-encoding genes *ampC, bla*_*SHV*_*, bla*_*OXA*_*, bla*_*CARB*_*, bla*_*TEM*_*, bla*_*CTX-M*_*, bla*_*CTX-M-1*_ group, *bla*_*CTX-M-2*_ group, *bla*_*CTX-M-8*_ group, *bla*_*CTX-M-9*_ group, *bla*_*CTX-M-15*_ group and *bla*_*CTX-M-25*_ group using primers described previously [26–28]. To set up PCR assays for *β*-lactam-encoding genes, a total of 25 µL reaction mixture consisted of 12.5 µL of the PCR Master Mix (AmpliTaq Gold® DNA Polymerase, 0.05 units/L, Gold buffer, 930 mM Tris/HCl pH 8.05, 100 mM KCl, 0.4 mM of each dNTP, and 5 mM MgCl2), 10 µM of each primer, 2 µL of template DNA and 8.5 µL nuclease-free water. The expected PCR product sizes are listed in Supplementary Table S1.

### Data analysis

Statistical analysis was carried out using Microsoft Excel 2016 (Microsoft Corporation, Redmond, DC, USA) and Statistical Package for the Social Sciences v. 26 (IBM Corporation, Armonk, NY, USA). The sequenced 16S rRNA gene of the representative isolates were aligned to nucleotide sequences available in GenBank and identified by comparing them with those available in the National Center for Biotechnology Information database (NCBI) using BLASTn (http://www.ncbi.nlm.nih.gov/BLAST/). Heatmap plots of the virulence and antibiotic resistance profiles were generated using ChipPlot (https://www.chiplot.online/#).

## Results

### Identification of d *E. coli*

In this study, nonduplicated (one isolate per sample) isolates of *E. coli* were recovered from 53 fecal samples. All 53 *E. coli* isolates were confirmed by the *uidA* gene PCR assay. However, the two isolates did not possess the *uidA* gene. A high (64.9%) incidence of *E. coli* was observed in sheep samples and a comparatively low occurrence was observed in goats (35.1%).

### Nucleotide sequence identity

The 16S rRNA gene sequence analysis of the *E. coli* isolates (n = 3) revealed a high percentage of nucleotide similarity (96.4 to 97.5%) to the reference GenBank sequences of the *E. coli* strains. The BLASTn results of 16S rRNA gene *E. coli* detected in this study (GenBank accession number: OR123648, OR123649 and OR123650) confirmed that it matches with relevant *E. coli* species on the NCBI database (GenBank accession numbers: KY458548.1, CP041429.1 (STEC367) and CP063518.1).

### Detection of virulence genes in *E. coli*

Six virulence genes were screened among the 53 *E. coli* isolates identified by PCR. Approximately 14 of the isolates were considered to belong to the *E. coli* O177 serogroup, as they contained the *wzy* gene. The Shiga toxin gene *stx2* (100%) was the most commonly detected gene, followed by *stx1* (47.2%), *vir* (18.9%) and (16.9%) *eaeA*, while *lt* and *aafII* were not detected. The majority (43.4%) of the isolates carried a combination of *stx2* + *stx1*, 5.7% carried a combination of *stx1* + *stx2* + *eaeA*, 22.6% harbored a mix of *stx1* + *stx2* + *eaeA* genes and 7.5% of the isolates possessed a mix of *stx1* + *stx2* + *vir* genes.

### Antibiotic susceptibility and resistance genes detected in *E. coli* isolates

Antimicrobial susceptibility tests revealed that all 53 *E. coli* isolates were resistant to nalidixic acid and ampicillin. Most of the isolates also showed resistance to erythromycin (66.04%; n = 37), colistin sulfate (43.4%; n = 23), chloramphenicol (9.4%; n = 5) and ciprofloxacin (1.9%; n = 1). In contrast, all isolates were susceptible to streptomycin. It was observed that 94.1% (n = 43/53) of the *E. coli* isolates were resistant to at least three classes of antibiotics and were all considered MDR.

**Fig. 1:**
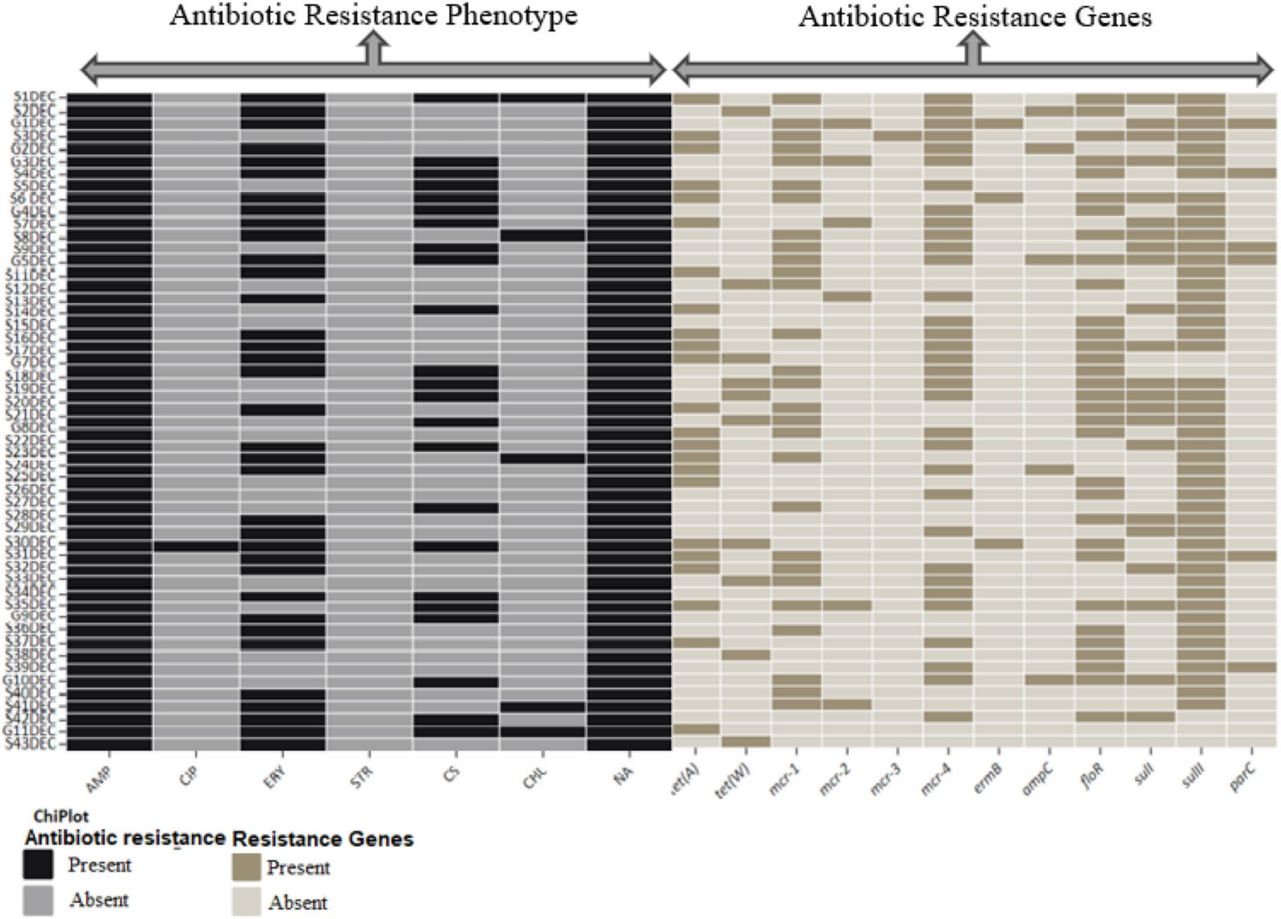
Heatmap showing the antibiotic resistance profiles (phenotype and genotype) from the *E. coli* isolated from fecal samples of sheep and goats. Black indicates the presence of an antibiotic resistance phenotype, while brown represents the presence of antibiotic resistance genes. https://www.chiplot.online/

The *E. coli* isolates (n = 53) from sheep and goat fecal samples harbored the antibiotic resistance genes *sul*II 46 (86.8%), *mcr-*4 33 (62.3%), *flo*R 33 (62.3%), *mcr-*1 28 (52.8%), *tet*(A) 23 (43.4%), *sul*I 22 (41.5%), *tet*(O) 11 (20.8%), *tet*(W) 10 (18.9%), *parC* 6 (11.3%), *mcr-2* 6 (11.3%), *ampC* 5 (9.4%), *qnrS* 5 (9.4%), and *ermB* 3 (5.7%). Meawhile the *tet(X), tet(P), ermB, mcr-3, mcr-5, catI, catII, catIII, catIV, sulIII, qnrA,* while *qnrD* genes were not detected in all 53 isolates (Table 2). Most (28.3%) of the isolates carried a combination of *mcr-1* + *mcr-4* + *sulII*, while 20.8% and 3.8% possessed *tet(A)* + *mcr-1* + *mcr-4* + *sulII* and a combination of *tet(W)9* + *mcr-1* + *floR* + *sulII* resistance genes, respectively. In this study, correlations between phenotypic antibiotic resistance patterns and antibiotic resistance genes encoding four antibiotics, nalidixic acid, erythromycin, chloramphenicol, ampicillin, and colistin sulfate, were observed in the isolates (Fig. 1).

**Table 2:** Antibiotic resistance phenotypes, genotype and prevalence of *β*-lactamase-encoding genes in *E. coli* isolates

No	Isolate codes	Resistance phenotype	Resistance genotype	β-lactamase-encoding genes
1	S1DEC*	AMP, ERY, CS, CHL, NA	*tet*(A), *mcr-*1, *flo*R, *sul*I, *sul*II	*blaSHV, CARB, TEM, CTX-M, CTX-M-2, CTX-M-9, CTX-M-25, CTX-M-15*
2	S2DEC	AMP, ERY, NA	*tet*(W), *mcr-*4, *amp*C, *flo*R, *sul*II	CTX-M-15
3	G1DEC	AMP, ERY, CS, NA	*mcr-*1, *mcr-*2, *mcr-*4, *erm*B, *sul*I, *sul*II, *par*C	*TEM, CTX-M, CTX-M-2, CTX-M-9 CTX-M-25, CTX-M-15*
4	S3DEC	AMP, NA	*tet*(A), *mcr-*1, *mcr-*3, *mcr-*4, *flo*R, *sul*I, *sul*II	*SHV, CTX-M-25*
5	G2DEC*	AMP, ERY, NA	*tet*(A), *mcr-*1, *mcr-*4, *amp*C, *sul*II	*TEM, CTX-M-2, CTX-M-9, CTX-M-25*
6	G3DEC	AMP, ERY, CS, NA	*mcr-*1, *mcr-*2, *mcr-*4, *flo*R, *sul*I, *sul*II	–
7	S4DEC*	AMP, NA	*flo*R, *sul*II, *par*C	*SHV, CTX-M, CTX-M-9, CTX-M-25,* CTX-M-15
8	S5DEC*	AMP, CS, NA	*tet*(A), *mcr-*1, *mcr-*4,	*CARB, CTX-M, CTX-M-2, CTX-M-9, CTX-M-25*
9	S6 DEC*	AMP, ERY, CS, NA	*tet*(A), *mcr-*1, *erm*B, *flo*R, *sul*I, *sul*II	*SHV, TEM, CTX-M-2, CTX-M-9, CTX-M-25,* CTX-M-15
10	G4DEC	AMP, ERY, NA	*mcr-*4, *flo*R, *sul*II	–
11	S7DEC	AMP, ERY, NA	*tet*(A), *mcr-*2, *mcr-*4, *sul*I, *sul*II	*TEM, TEM, CTX-M, CTX-M-25*
12	S8DEC	AMP, ERY, CHL, NA	*mcr-*1, *mcr-*4, *flo*R, *sul*I, *sul*II	*SHV, CARB, CTX-M-2, CTX-M-9,* CTX-M-15
13	S9DEC	AMP, NA	*mcr-*1, *mcr-*4, *sul*I, *sul*II, *par*C	*TEM, CTX-M-9, CTX-M-25*
14	G5DEC	AMP, ERY, NA	*mcr-*1, *mcr-*4, *amp*C, *flo*R, *sul*I, *sul*II *par*C	–
15	S11DEC*	AMP, ERY, NA	*tet*(A), *mcr-*1, *sul*II	*SHV, TEM, CTX-M, CTX-M-2, CTX-M-9, CTX-M-25,* CTX-M-15
16	S12DEC	AMP, NA	*tet*(W), *mcr-*1, *flo*R, *sul*II	–
17	S13DEC	AMP, ERY,	*mcr-*2, *mcr-*4, *sul*II	*CARB, CTX-M, CTX-M-25*
18	S14DEC	AMP, CS, NA	*tet*(A), *sul*I, *sul*II	*TEM, CTX-M-9, CTX-M-25,* CTX-M-15
19	S158DEC	AMP, NA	*mcr-*4, *flo*R, *sul*II	*CTX-M-9*
20	S15DEC*	AMP, ERY, CS, NA	*tet*(A), *mcr-*1, *mcr-*4, *flo*R, *sul*II	*CARB, TEM, CTX-M-9, CTX-M-25,* CTX-M-15
21	S16DEC	AMP, ERY, CS, NA	*tet*(A), *mcr-*4, *flo*R, *sul*I, *sul*II	CTX-M-15
22	S17DEC	AMP, ERY, NA	*tet*(A), *tet*(W), *mcr-*4, *flo*R,	*TEM, CTX-M, CTX-M-25*
23	G7DEC	AMP, ERY, NA	*mcr-*1, *mcr-*4,	*CARB, CTX-M-25,* CTX-M-15
24	S18DEC	AMP, CS, NA	*tet*(W), *mcr-*1, *mcr-*4, *flo*R, *sul*I, *sul*II	*CTX-M,* CTX-M-15
25	S19DEC	AMP, NA	*tet*(W), *mcr-*4, *flo*R, *sul*I, *sul*II	–
26	S20DEC	AMP, ERY, CS, NA	*tet*(A), *mcr-*1, *flo*R, *sul*I, *sul*II	*SHV, CTX-M-2, CTX-M-9, CTX-M-25*
27	S21DEC	AMP, CS, NA	*tet*(W), *mcr-*1, *flo*R, *sul*I, *sul*II	*CTX-M-25*
28	G8DEC	AMP, CS, NA	*tet*(A), *mcr-*1, *mcr-*4, *flo*R, *sul*II	*CARB, CTX-M*
29	S22DEC	AMP, ERY, NA	*tet*(A), *mcr-*4, *sul*I, *sul*II	*CTX-M-9, CTX-M-25*
30	S23DEC	AMP, ERY, CS, CHL, NA	*tet*(A), *mcr-*1, *sul*II	*CTX-M-25*
31	S24DEC	AMP, ERY, CS, NA	*tet*(A), *mcr-*4, *amp*C, *sul*II	–
32	S25DEC*	AMP, NA	*tet*(A), *flo*R, *sul*II	*SHV, CARB, CTX-M-2, CTX-M-9, CTX-M-25*
33	S26DEC	AMP, ERY, CS,	*mcr-*4, *flo*R, *sul*II	*TEM,* CTX-M-15
34	S27DEC	AMP, CS, NA		*CTX-M-9,* CTX-M-15
35	S28DEC	AMP, ERY, CS, NA	*flo*R, *sul*I, *sul*II	*CTX-M-9,* CTX-M-15
36	S29DEC*	AMP, ERY, CS, NA	*mcr-*4, *sul*I, *sul*II	*TEM, CTX-M-2, CTX-M-9, CTX-M-25*
37	S30DEC	AMP, CIP, ERY, NA	*tet*(A), *tet*(W), *erm*B, *flo*R, *sul*II	–
38	S31DEC	AMP, ERY, NA	*tet*(A), *flo*R, *sul*II, *par*C	*CTX-M-9,* CTX-M-15
39	S32DEC	AMP, ERY, CS, NA	*tet*(A), *mcr-*1, *mcr-*4, *sul*I, *sul*II	–
40	S33DEC*	AMP, CS, NA	*tet*(W), *mcr-*1, *mcr-*4, *sul*II	*CARB, CTX-M-2, CTX-M-9, CTX-M-25*
41	S34DEC	AMP, ERY, NA	*mcr-*4, *sul*II	*TEM,* CTX-M-15
42	S35DEC	AMP, ERY, NA	*tet*(A), *mcr-*1, *mcr-*2, *mcr-*4, *flo*R, *sul*I, *sul*II	*SHV, CTX-M*
43	G9DEC*	AMP, ERY, NA	*sul*II	*CARB, CTX-M-2, CTX-M-9, CTX-M-25*
44	S36DEC	AMP, ERY, NA	*mcr-*1, *flo*R, *sul*II	*SHV*
45	S37DEC*	AMP, ERY, NA	*tet*(A), *mcr-*4, *flo*R, *sul*II	*CTX-M-2, CTX-M-9, CTX-M-25*
46	S38DEC	AMP, NA	*tet*(W), *flo*R, *sul*II	CTX-M-15
47	S39DEC	AMP, ERY, NA	*mcr-*4, *flo*R, *sul*II, *par*C	*SHV, CARB, TEM, CTX-M, CTX-M-25*
48	G10DEC	AMP, NA	*mcr-*1, *mcr-*4, *amp*C, *flo*R, *sul*I, *sul*II	–
49	S40DEC*	AMP, ERY, NA	*mcr-*1 *sul*II	*SHV, CTX-M, CTX-M-2, CTX-M-9, CTX-M-25,* CTX-M-15
50	S41DEC	AMP, ERY, CHL, NA	*mcr-*1, *mcr-*2, *sul*II	*TEM, CTX-M-9,* CTX-M-15
51	S42DEC	AMP, ERY, NA	*mcr-*4, *flo*R, *sul*I,	*CTX-M, CTX-M-2, CTX-M-9*
52	G11DEC*	AMP, ERY, CS, NA	*tet*(A)	*CARB, CTX-M-9, CTX-M-25,* CTX-M-15
53	S43DEC*	AMP, ERY, CS, CHL, NA	*tet*(W)	*SHV, TEM, CTX-M-2, CTX-M-25*

*S* streptomycin, *CIP* ciprofloxacin, *NA* nalidixic acid, *E* erythromycin, *AMP* ampicillin, *CS* colistin sulfate, *CHL* chloramphenicol

*ESBL-producing *E. coli*

### Prevalence of ESBL-producing *E. coli* isolates

The phenotypes of ESBL-producing *E. coli* were recovered from 15 samples, of which 3/15 (20%) were from goats and 12/15 (80%) were from sheep. Therefore, 38/52 (71.7%) *E. coli* isolates were excluded from further screening for ESBL. Of the 15 ESBL-positive *E. coli* isolates, 15/15 (100%) harbored both the *bla*_*CTX-M-9*_ and *bla*_*CTX-M-25*_ genes. Meanwhile, 13/15 (81.3%) harbored the *bla*_*CTX-M-2*_ gene, 10/15 (62.5%) *bla*_*SHV*_, 7/15 (43.8%) *bla*_*CARB*_, 7/15 (43.8%) *bla*_*TEM*_, 7/15 (13.2%) *bla*_*CTX-M-15*_ and 5/15 (31.3%) *bla*_*CTX-M*_. All the isolates were negative for the *bla*_*OXA*_, *bla*_*CTX-M-*1_, and *bla*_*CTX-M-8*_ groups (Table 2). Thirteen (81.3%) of the ESBL-producing *E. coli* isolates were considered MDR.

### Coexistence of ESBL-producing *E. coli* and virulence genes

**Fig. 2:**
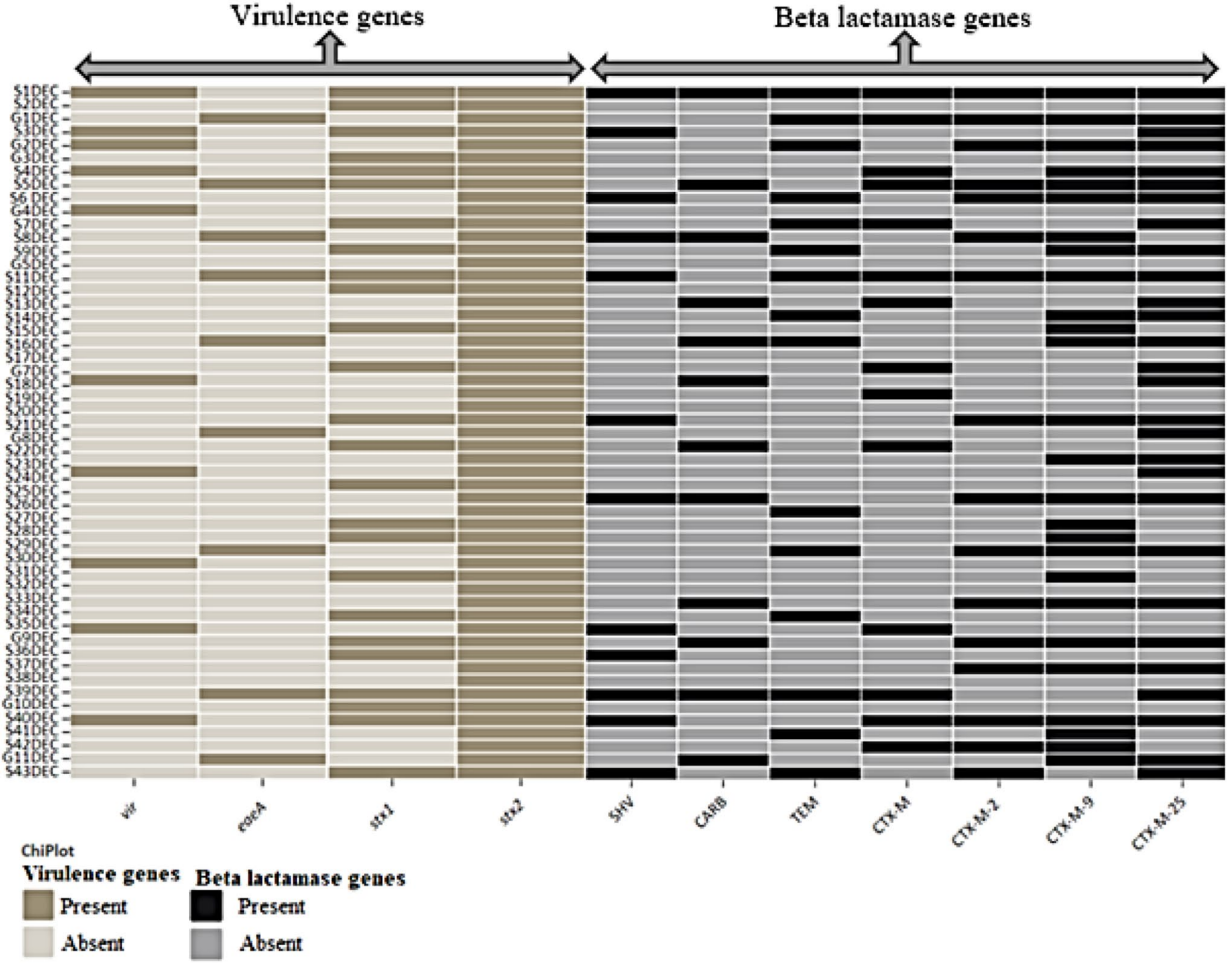
Heatmap showing the clustering of the beta-lactamase and virulence genes in *E. coli* strains isolated from fecal samples of sheep and goats. Dark and light gray colors indicate the presence and absence of the genes, respectively. https://www.chiplot.online/

Only 16 isolates consisted of one virulence gene (*stx2*) and two *bla* genes (*bla*_*CTX-M-9*_ and *bla*_*CTX-M-25*_ groups), while six isolates harbored two virulence genes (*stx1* and *stx2*) and two *bla* genes (*bla*_CTX-M-9_ and *bla*_*CTX-M-25*_ groups). One isolate (S1DEC) harbored all the *bla* genes screened in this study and three virulent genes (*stx1*, *stx2* and *vir*). Another isolate (S4DEC) possessed four *vir* + *eaeA* + *stx1* + *stx2* virulence genes and three *bla* genes (bla_CTX-M-9_ + *bla*_*CTX-M-25*_ + *bla*_*CTX-M-15*_) (Fig. 2). The *bla*_*CTX-M-25*_ and bla_*CTX-M-9*_ groups, as well as *stx1* and *stx2*, had the most genes detected in the *E. coli* strains isolated in this study.

## Discussion

This study investigated the existence of STEC in sheep and goats as well as the isolates’ capacity to produce extended-spectrum beta-lactamase (ESBL) and the Shiga toxin. Cattle, goats and sheep serve as natural reservoirs for *E. coli* in their guts [29]. As a result of their virulence factors, diarrheagenic *E. coli* strains cause diarrhea in both animal and human hosts [30]. In the present study, the *uidA* gene was used to confirm the isolates as *E. coli*. All the *E. coli* strains obtained from this study were positive for at least one of the virulence genes.

In this study, the Shiga toxin gene *stx2* (100%) was the most detected gene, followed by *stx1* (47.2%), as well as enteroinvasive *E. coli* (*vir*) 18.9%, and enterohemorrhagic *E. coli* (*eaeA*) 16.9% virulence genes. This observation was different to the findings of the previous studies conducted in South Africa, where *stx1* had the highest prevalence in human [31]. Different findings were reported by Dela et al. [32] in Ghana, whereby none of the *E. coli* isolates tested positive for *stx1, stx2* and *eaeA*. The combination of *stx2/stx1* genes was observed in (43.4%) of the isolates. This is the most relevant finding because it suggests that STEC strains are circulating among sheep and goats. Furthermore, strains harboring the *stx2* gene are considered more virulent [33–35]. Additionally, the strain carrying *stx1* may cause diarrhea in immunocompromised individuals [33]. Nine isolates of *E. coli* (16.9%) consisted of the *eaeA* gene. This is a gene of enteropathogenic *E. coli* (EPEC) that is necessary for intimate attachment to epithelial cells [36]. These findings are higher than those reported in China, where 9.5% of *E. coli* strains harbored this gene [37]. The *eae*A gene encodes an outer membrane that mediates the adhesion of STEC/EPEC to the intestinal epithelium [34]. Enterotoxigenic *E. coli* (ETEC) strains expressing the *eaeA* gene are potentially capable of causing attaching and effacing lesions in humans [38]. In this study, the *eaeA* gene was detected in 9.4% of the *E. coli* isolates. The study conducted by Ercoli et al. [39] in Italy reported a high prevalence of this gene (50%) in swine fecal samples, in South Africa Ramatla et al. [40], reported a high prevalence of this gene (14%) in *Rattus* species fecal samples. Variations in detection and isolation methods of STEC strains may also contribute to differences in prevalence rates [39, 41]. The ETEC strains that contain the *lt* and *aafII* genes were not detected in this study. These results are in agreement with the reports of other studies from South Africa and Iran, where these genes were not detected in children with acute diarrhea samples and riding horse samples, respectively [19, 42]. However, the observations differ from the study conducted in Turkey, whereby the *lt* gene was detected in one isolate from clinical mastitis bovine milk [38].

Globally, diarrheagenic bacterial pathogens are becoming increasingly resistant to antimicrobials, especially in less developed areas [19, 43]. Resistance to a majority of antibiotics has been developing as a result of improper use and overuse of antimicrobials [44]. In this study, all *E. coli* strains were resistant to nalidixic acid and ampicillin. The majority of the isolates also showed resistance to erythromycin (66.04%), colistin sulfate (43.4%), chloramphenicol (9.4%) and ciprofloxacin (1.9%). Resistance to antibiotics, especially chloramphenicol, ampicillin and tetracycline [39] and fluoroquinolones [15], has been reported in previous studies on *E. coli* isolates. The correlation between phenotypic and antimicrobial resistance genes encoding four antibiotics, nalidixic acid, erythromycin, chloramphenicol, ampicillin and colistin sulfate, was observed in this study.

Antimicrobial resistance genes allow bacteria to survive and resist the effects of antibiotics [45]. These genes can be passed on to future generations of bacteria, making them more resistant to antibiotics and leading to the development of superbugs [46]. This is a serious health concern, as it reduces the effectiveness of antibiotics and can result in increased mortality rates. The majority of the isolates harbored multiple resistance genes and some contained up to six antibiotic resistance genes. These genes were associated with resistance to multiple classes of antibiotics, including erythromycin, colistin sulfate, chloramphenicol, and ciprofloxacin. In the current study, 94.1% (n = 43/53) of the *E. coli* isolates were resistant to multiple antibiotics. This suggests that antibiotic resistance is widespread in *E. coli* isolates. The MDR bacteria are resistant to multiple antibiotics, so infections caused by them can be difficult to treat. This can lead to prolonged hospital stays, increased medical costs and even death in some cases [47].

Antibiotic resistance genes such as *sulII, mcr-4, floR*, and *mcr-1* were the most frequently detected in 46 (86.8%), 33 (62.3%), 33 (62.3%) and 28 (52.8%) *E. coli* isolates, respectively. The colistin *mcr-1, mcr-2* and *mcr-4* resistance genes were detected in this study. This is because colistin is utilized to stimulate animal growth [48] and it can be found in concentrations that are too high for humans to consume safely in some countries. Despite having only 6% of the world’s population, Brazil, China, India, Russia and South Africa together account for 13% of colistin use, according to a statistical analysis from 2000 to 2010 [49]. Due to its success in treating infections that are resistant to antibiotics, colistin use has increased dramatically in these nations in recent years.

Resistance to antibiotics belonging to the beta-lactam group has been increasing recently, probably due to the high prescription of these antibiotics [50]. There is also an increasing recognition of livestock carrying extended-spectrum beta-lactamase-producing *Escherichia coli* as a potential source of the spread of these microorganisms to humans, where livestock and humans share the same residence [51]. This increase in resistance is due to the overuse of these antibiotics. As a result, these antibiotics become less effective in treating bacterial infections. In our study, 15 (28.8%) ESBL-*E. coli* isolates were identified. As expected, of the 15 ESBL-*E. coli* isolates, *bla*_*CTX*_ was the most prevalent gene in our ESBL-positive isolates. The *bla*_*CX-M-9*_ and *bla*_*CX-M-25*_ genes were found to be the most prevalent ESBL-encoding genes in *E. coli* isolates, and their prevalence was 100%. The *bla*_*CTX-M-15*_, which remains the most widely disseminated genotype worldwide, was detected in seven ESBL-producing *E. coli* isolates. There was a low frequency of *E. coli* carrying the *bla*_*CTX-M*_ gene (5: 31.3%) among our isolates, which is similar to what has been reported by Nakhaei et al. [52] in Iran. Differences in animal husbandry practices and the use of antibiotics in food animals may also play a role in the varying rates. Thus, there is a need for increased surveillance and preventive measures to reduce the spread of antibiotic resistance.

## Conclusion

This is the first study to report the prevalence of STEC, including the ESBL producing *E. coli* (STEC) in sheep and goats in South Africa. The *E. coli* strains from this study displayed high levels of resistance traits against the erythromycin and colistin sulphate antibiotic groups. This is especially concerning since *E. coli* is a common commensal in humans and is known to cause severe gastrointestinal infections. An interesting point to highlight from the results of this study is the presence of STEC isolates expressing a combination of the *wzy/eaeA/stx1/stx2* virulent genes and three *bla* genes (CTX-M-9/*CTX-M-25/CTX-M-15*). The high proportion of the *mcr* and *stx2* gene detected in *E. coli* represents a serious public-health threat. This highlights the need for increased vigilance and proactive measures to ensure the safety of the population from these potentially harmful pathogens.

## Supplementary Information

Below is the link to the electronic supplementary material.

Supplementary file1 (DOCX 30 kb)

## Data Availability

The data and materials of the study will be available from the corresponding author on reasonable request. The sequences of the two strains analyzed were deposited in the National Library of Medicine, National Center for Biology Information (NCBI), GenBank nucleotide sequence database. The accession numbers assigned as OR123648 (https://www.ncbi.nlm.nih.gov/nuccore/OR123648), OR123649 (https://www.ncbi.nlm.nih.gov/nuccore/OR123649) and OR123650 (https://www.ncbi.nlm.nih.gov/nuccore/OR123650).

## Abbreviations

STEC: Shiga toxin producing *Escherichia coli*
EHEC: Enterohemorrhagic *E. coli*
ETEC: Enterotoxigenic *E. coli*
EIEC: Enteroinvasive *E. coli*
EAEC: Enteroaggregative *E. coli*
AMR: Antimicrobial resistance
MDR: Multidrug resistant
CLSI: Clinical and laboratory standards institute
WHO: World Health Organization
DNA: Deoxyribonucleic acid
ESBL: Extended spectrum beta lactamase
BLAST: Basic local alignment search tool
SMA: Sorbitol MacConkey agar
NCBI: National center for biotechnology information
PCR: Polymerase chain reaction

## References

[1] Allocati N, Masulli M, Alexeyev MF, Di Ilio C (2013) *Escherichia coli* in Europe: an overview. Int J Environ Res Public Health 10(12):6235–6254 24287850 10.3390/ijerph10126235 PMC3881111

[2] Nada HG, El-Tahan AS, El-Didamony G, Askora A (2023) Detection of multidrug-resistant Shiga toxin-producing *Escherichia coli* in some food products and cattle faeces in Al-Sharkia, Egypt: one health menace. BMC Microbiol 23(1):127 37173663 10.1186/s12866-023-02873-2 PMC10176883

[3] Etcheverria AI, Padola NL (2013) Shiga toxin-producing *Escherichia coli*: factors involved in virulence and cattle colonization. Virulence 4(5):366–372 23624795 10.4161/viru.24642 PMC3714128

[4] Caprioli A, Tozzoli R, Morabito S, Strockbine NA (2012) Multicenter evaluation of a sequence-based protocol for subtyping Shiga toxins and standardizing Stx nomenclature. J Clin Microbiol 50(9):2951–2963 22760050 10.1128/JCM.00860-12 PMC3421821

[5] Melton-Celsa AR (2014) Shiga toxin (Stx) classification, structure and function. Microbiol Spectr. 10.1128/microbiolspec.EHEC-0024-2013 25530917 10.1128/microbiolspec.EHEC-0024-2013 PMC4270005

[6] Shahzad A, Ullah F, Irshad H, Ahmed S, Shakeela Q, Mian AH (2021) Molecular detection of Shiga toxin-producing *Escherichia coli* (STEC) O157 in sheep, goats, cows and buffaloes. Mol Biol Rep 48(8):6113–6121 34374895 10.1007/s11033-021-06631-3

[7] Martínez-Vázquez AV, Vázquez-Villanueva J, Leyva-Zapata LM, Barrios-García H, Rivera G, Bocanegra-García V (2021) Multidrug resistance of *Escherichia coli* strains isolated from Bovine Feces and Carcasses in Northeast Mexico. Front Vet Sci 8:643802 33969038 10.3389/fvets.2021.643802 PMC8102688

[8] Mandujano A, Cortés-Espinosa DV, Vásquez-Villanueva J, Guel P, Rivera G, Juárez-Rendón K, Cruz-Pulido WL, Aguilera-Arreola G, Guerrero A, Bocanegra-García V (2023) Extended-spectrum *β*-Lactamase-producing *Escherichia coli* isolated from food-producing animals in Tamaulipas. Mexico Antibiotics 12:1010 37370329 10.3390/antibiotics12061010 PMC10294937

[9] Costa WF, Paranhos R, Mello MP, Pic˜ao RC, Laport MS (2023) Occurrence of extended-spectrum β-lactamases-producing *Escherichia coli* isolates over gradient pollution in an urban tropical estuary. Environ Microbiol 2023:1–8 10.1111/1462-2920.16435 37280775

[10] Hesp A, Veldman K, van der Goot J, Mevius D, van Schaik G (2019) Monitoring antimicrobial resistance trends in commensal *Escherichia coli* from livestock, the Netherlands, 1998 to 2016. Euro Surveill 24(25):1800438 31241037 10.2807/1560-7917.ES.2019.24.25.1800438 PMC6593905

[11] Singer RS, Finch R, Wegener HC, Bywater R, Walters J, Lipsitch M (2003) Antibiotic resistance—the interplay between antibiotic use in animals and human beings. Lancet Infect Dis 3(1):47–51 12505035 10.1016/s1473-3099(03)00490-0

[12] Laube H, Friese A, Von Salviati C, Guerra B, Käsbohrer A, Kreienbrock L, Roesler U (2013) Longitudinal monitoring of extended-spectrum-beta-lactamase/AmpC-producing *Escherichia coli* at German broiler chicken fattening farms. Appl Environ Microbiol 79(16):4815–4820 23747697 10.1128/AEM.00856-13 PMC3754693

[13] Xexaki A, Papadopoulos DK, Alvanou MV, Giantsis IA, Papageorgiou KV, Delis GA, Economou V, Kritas SK, Sossidou EN, Petridou E (2023) Prevalence of antibiotic resistant *E. coli* strains isolated from farmed broilers and hens in Greece, based on phenotypic and molecular analyses. Sustainability 15(12):9421

[14] Haddadin RN, Collier PJ, Haddadin S (2023) Phenotypic ESBL and non-phenotypic ESBL isolates of *Klebsiella pneumoniae* exhibit differing responses to induced antimicrobials resistance and subsequent antibiotic cross-resistance. J Appl Microbiol 134(2):p.lxac082 10.1093/jambio/lxac082 36724268

[15] Hawari AD, Al-Dabbas F (2008) Prevalence and distribution of mastitis pathogens and their resistance against antimicrobial agents in dairy cows in Jordan. Am J Anim Vet Sci 3:36–39

[16] Elbadawi HS, Elhag KM, Mahgoub E, Altayb HN, Ntoumi F, Elton L, McHugh TD, Tembo J, Ippolito G, Osman AY, Zumla A (2021) Detection and characterization of carbapenem resistant Gram-negative bacilli isolates recovered from hospitalized patients at Soba University Hospital. Sudan BMC Microbiol 21(1):1–9 10.1186/s12866-021-02133-1 PMC8094518 33947325

[17] Sajeev S, Hamza M, Rajan V, Vijayan A, Sivaraman GK, Shome BR, Holmes MA (2023) Resistance profiles and genotyping of extended-spectrum beta-lactamase (ESBL)-producing and non-ESBL-producing *E. coli* and *Klebsiella* from retail market fishes. Infect Genet Evol 2023:105446 10.1016/j.meegid.2023.105446 37245778

[18] World Health Organization (WHO) (2021) WHO integrated global surveillance on ESBL-producing *E. coli* using a “One Health” approach: implementation and opportunities. World Health Organization, Geneva

[19] Omolajaiye SA, Afolabi KO, Iweriebor BC (2020) Pathotyping and antibiotic resistance profiling of *Escherichia coli* isolates from children with acute diarrhea in amatole district municipality of Eastern Cape. South Africa Biomed Res Int 18(2020):1–10 10.1155/2020/4250165 PMC7691003 33294442

[20] Mariam SH (2021) Isolation and characterization of gram-negative bacterial species from pasteurized dairy products: potential risk to consumer health. J Food Qual 2021:1

[21] Wolde A, Deneke Y, Sisay T, Mathewos M (2022) Molecular characterization and antimicrobial resistance of pathogenic *Escherichia coli* strains in children from Wolaita Sodo, Southern Ethiopia. J Trop Med 2022:9166209 35846070 10.1155/2022/9166209 PMC9279085

[22] Bauer AW, Kirby WM, Sherris JC, Turck M (1966) Antibiotic susceptibility testing by a standardized single disk method. Am J Clin Pathol 45:493–496 5325707

[23] Clinical and Laboratory Standards Institute (CLSI) (2018) Performance standards for antimicrobial susceptibility testing. Clinical and Laboratory Standards Institute, Wayne

[24] Ramatla T, Taioe MO, Thekisoe OM, Syakalima M (2019) Confirmation of antimicrobial resistance by using resistance genes of isolated *Salmonella* spp. in chicken houses of North West, South Africa. World Vet J 9(3):158–165

[25] Jousset AB, Bernabeu S, Bonnin RA, Creton E, Cotellon G, Sauvadet A, Naas T, Dortet L (2019) Development and validation of a multiplex polymerase chain reaction assay for detection of the five families of plasmid-encoded colistin resistance. Int J Antimicrob Agents 53(3):302–309 30395987 10.1016/j.ijantimicag.2018.10.022

[26] Liu G, Ding L, Han B, Piepers S, Naqvi SA, Barkema HW, Ali T, De Vliegher S, Xu S, Gao J (2018) Characteristics of *Escherichia coli* isolated from bovine mastitis exposed to subminimum inhibitory concentrations of cefalotin or ceftazidime. Biomed Res Int 2018:4301628 30515397 10.1155/2018/4301628 PMC6236695

[27] Ramatla T, Mileng K, Ndou R, Mphuti N, Syakalima M, Lekota KE, Thekisoe OM (2022) Molecular detection of integrons, colistin and β-lactamase resistant genes in Salmonella enterica serovars enteritidis and typhimurium isolated from chickens and rats inhabiting poultry farms. Microorganisms 10(2):313 35208768 10.3390/microorganisms10020313 PMC8876313

[28] Gundran RS, Cardenio PA, Villanueva MA, Sison FB, Benigno CC, Kreausukon K, Pichpol D, Punyapornwithaya V (2019) Prevalence and distribution of bla CTX-M, bla SHV, bla TEM genes in extended-spectrum β-lactamase-producing *E. coli* isolates from broiler farms in the Philippines. BMC Vet Res 15:1–8 31277658 10.1186/s12917-019-1975-9 PMC6612079

[29] Persad AK, Lejeune JT (2015) Animal reservoirs of Shiga toxin-producing *Escherichia coli*. In: Hovde CJ, Sperandio V (eds) Enterohemorrhagic *Escherichia coli* and other Shiga toxin-producing *E. coli*. ASM Press, Washington, pp 211–230

[30] Ali DA, Tesema TS, Belachew YD (2021) Retracted article: molecular detection of pathogenic *Escherichia coli* strains and their antibiogram associated with risk factors from diarrheic calves in Jimma Ethiopia. Sci Rep 11(1):1–15 34257358 10.1038/s41598-021-93688-6 PMC8277816

[31] Karama M, Mainga AO, Cenci-Goga BT, Malahlela M, El-Ashram S, Kalake A (2019) Molecular profiling and antimicrobial resistance of Shiga toxin-producing *Escherichia coli* O26, O45, O103, O121, O145 and O157 isolates from cattle on cow-calf operations in South Africa. Sci Rep 9(1):1–15 31417098 10.1038/s41598-019-47948-1 PMC6695430

[32] Dela H, Egyir B, Majekodunmi AO, Behene E, Yeboah C, Ackah D, Bongo RN, Bonfoh B, Zinsstag J, Bimi L, Addo KK (2022) Diarrhoeagenic *E. coli* occurrence and antimicrobial resistance of extended spectrum beta-lactamases isolated from diarrhoea patients attending health facilities in Accra, Ghana. PLoS ONE 17(5):e0268991 35617316 10.1371/journal.pone.0268991 PMC9135277

[33] Farrokh C, Jordan K, Auvray F, Glass K, Oppegaard H, Raynaud S, Thevenot D, Condron R, De Reu K, Govaris A, Heggum K (2013) Review of Shiga-toxin-producing *Escherichia coli* (STEC) and their significance in dairy production. Int J Food Microbiol 162(2):190–212 22939912 10.1016/j.ijfoodmicro.2012.08.008

[34] Montso PK, Mlambo V, Ateba CN (2019) The first isolation and molecular characterization of Shiga toxin-producing virulent multi-drug resistant atypical enteropathogenic *Escherichia coli* O177 serogroup from South African Cattle. Front Cell Infect Microbiol 9:333 31608246 10.3389/fcimb.2019.00333 PMC6769085

[35] Karam M, Mainga AO, Cenci-Goga BT, Malahlela M, El-Ashram S, Kalake A (2019) Molecular profiling and antimicrobial resistance of Shiga toxin-producing *Escherichia coli* O26, O45, O103, O121, O145 and O157 isolates from cattle on cow-calf operations in South Africa. Sci Rep 9(1):11930 31417098 10.1038/s41598-019-47948-1 PMC6695430

[36] Donnenberg MS, Tacket CO, James SP, Losonsky G, Nataro JP, Wasserman SS, Kaper JB, Levine MM (1993) Role of the eaeA gene in experimental enteropathogenic *Escherichia coli* infection. J Clin Investig 92(3):1412–1417 8376594 10.1172/JCI116717 PMC288285

[37] Yang X, Bai X, Zhang J, Sun H, Fu S, Fan R, He X, Scheutz F, Matussek A, Xiong Y (2020) *Escherichia coli* strains producing a novel Shiga toxin 2 subtype circulate in China. Int J Med Microbiol 310(1):151377 31757694 10.1016/j.ijmm.2019.151377

[38] Yarar M, Turkyilmaz S (2019) Investigation of antibiotic resistance and important virulence genes of *Escherichia coli* isolated from clinical mastitic bovine milk. Isr J Vet Med 74(2):74–81

[39] Ercoli L, Farneti S, Zicavo A, Mencaroni G, Blasi G, Striano G, Scuota S (2016) Prevalence and characteristics of verotoxigenic *Escherichia coli* strains isolated from pigs and pork products in Umbria and Marche regions of Italy. Int J Food Microbiol 232:7–14 27236076 10.1016/j.ijfoodmicro.2016.05.002

[40] Ramatla TA, Mphuthi N, Ramaili T, Taioe M, Thekisoe O, Syakalima M (2022) Molecular detection of zoonotic pathogens causing gastroenteritis in humans: *Salmonella* spp., *Shigella* spp. and *Escherichia coli* isolated from *Rattus* species inhabiting chicken farms in North West Province South Africa. S Afr Vet Assoc 93(2):63–69 10.36303/JSAVA.83 35934902

[41] Tseng M, Fratamico PM, Manning SD, Funk JA (2014) Shiga toxin-producing *Escherichia coli* in swine: the public health perspective. Anim Health Res Rev 15(1):63–75 24397985 10.1017/S1466252313000170 PMC4121380

[42] Reshadi P, Heydari F, Ghanbarpour R, Bagheri M, Jajarmi M, Amiri M, Alizade H, Badouei MA, Sahraei S, Adib N (2021) Molecular characterization and antimicrobial resistance of potentially human-pathogenic *Escherichia coli* strains isolated from riding horses. BMC Vet Res 17(1):1–9 33766016 10.1186/s12917-021-02832-x PMC7992949

[43] Heidary M, Momtaz H, Madani M (2014) Characterization of diarrheagenic antimicrobial resistant *Escherichia coli* isolated from pediatric patients in Tehran. Iran Iran Red Crescent Med J 16(4):e12329 24910786 10.5812/ircmj.12329 PMC4028759

[44] Rather IA, Kim BC, Bajpai VK, Park YH (2017) Self-medication and antibiotic resistance: crisis, current challenges and prevention. Saudi J Biol Sci 24(4):808–812 28490950 10.1016/j.sjbs.2017.01.004 PMC5415144

[45] Haenni M, Dagot C, Chesneau O, Bibbal D, Labanowski J, Vialette M, Bouchard D, Martin-Laurent F, Calsat L, Nazaret S, Petit F (2022) Environmental contamination in a high-income country (France) by antibiotics, antibiotic-resistant bacteria and antibiotic resistance genes: status and possible causes. Environ Int 159:107047 34923370 10.1016/j.envint.2021.107047

[46] García J, García-Galán MJ, Day JW, Boopathy R, White JR, Wallace S, Hunter RG (2020) A review of emerging organic contaminants (EOCs), antibiotic resistant bacteria (ARB) and antibiotic resistance genes (ARGs) in the environment: Increasing removal with wetlands and reducing environmental impacts. Bioresour Technol 307:123228 32247686 10.1016/j.biortech.2020.123228

[47] Van Duin D, Paterson DL (2016) Multidrug-resistant bacteria in the community: trends and lessons learned. Infect Dis Clin 30(2):377–390 10.1016/j.idc.2016.02.004 PMC5314345 27208764

[48] Kumar H, Chen BH, Kuca K, Nepovimova E, Kaushal A, Nagraik R, Bhatia SK, Dhanjal DS, Kumar V, Kumar A, Upadhyay NK (2020) Understanding of colistin usage in food animals and available detection techniques: a review. Animals 10:1892 33081121 10.3390/ani10101892 PMC7602861

[49] Laxminarayan R, Matsoso P, Pant S, Brower C, Røttingen JA, Klugman K, Davies S (2016) Access to effective antimicrobials: a worldwide challenge. Lancet 387(10014):168–175 26603918 10.1016/S0140-6736(15)00474-2

[50] Atta HI, Idris SM, Gulumbe BH, Awoniyi OJ (2022) Detection of extended spectrum beta-lactamase genes in strains of *Escherichia coli* and *Klebsiella pneumoniae* isolated from recreational water and tertiary hospital waste water in Zaria, Nigeria. Int J Environ Health Res 32(9):2074–2082 34151649 10.1080/09603123.2021.1940884

[51] Devi LS, Broor S, Chakravarti A, Chattopadhya D (2020) Livestock manure as potential reservoir of CTX-M type extended-spectrum *β-*lactamase producing *Escherichia coli* and *Klebsiella pneumoniae* associated with carbapenemase production. J Pure Appl Microbiol 14(1):171–181

[52] Nakhaei MM, Hashemi BM, Amel JS, Ghahraman M (2014) Genetic properties of blaCTX-M and blaPER β-lactamase genes in clinical isolates of Enterobacteriaceae by polymerase chain reaction. Iran J Basic Med Sci 17(5):378–383 24967067 PMC4069837

