# Supplementary material for: Molecular detection of Shiga toxin and extended-spectrum beta-lactamase (ESBL)-producing Escherichia coli isolates from sheep and goats
Source: Mol Biol Rep. 2024 Jan 2;51(1):57. doi: 10.1007/s11033-023-08987-0 (PMC10761393; doi:10.1007/s11033-023-08987-0)
Supplement: Supplementary file 1 — Supplementary file1 (DOCX 30 kb) [file 11033_2023_8987_MOESM1_ESM.docx]

**Molecular detection of** **Shiga toxin and extended-spectrum beta-lactamase (ESBL) -producing** ***Escherichia coli* isolates from sheep and goats**

Tsepo Ramatla^1^*, Tshepang Motlhaping^1^, Lara de Wet^1^, Prudent Mokgokong^1^, Oriel Thekisoe ^1^, Kgaugelo Lekota^1^

^1^Unit for Environmental Sciences and Management, North-West University, Potchefstroom, 2531, South Africa.

*Corresponding author: Tsepo Ramatla, Email: ra21205450@gmail.com

**Table 1.** List of antibiotic resistance genes primers used in this study.

| **Class** | **Target gene** | **Primer** | **Primer sequence (5´ → 3´)** | **Amplicon size (bp)** | **Annealing temp (°C)** | **References** |
| --- | --- | --- | --- | --- | --- | --- |
| **Tetracycline** | *tet*(A) | TETA-F  TETA-R | GCGCTNTATGCGTTGATGCA  ACAGCCCGTCAGGAAATT | 387 | 62 | Jiang et al., 2013 |
|  | *tet*(O) | TETO-F  TETO-R | ACGGARAGTTTATTGTATACC  TGGCGTATCTATAATGTTGAC | 171 | 60 | Aminov et al., 2001. |
|  | *tet*(X) | TETX-F  TETX-R | CCGACACGGAAGTTGAAGAA  CCTTGGTGAGATGCCATTAGC | 468 | 60 | Aminov et al., 2001. |
|  | *tet*(P) | TETP-F  TETP-R | CTTGGATTGCGGAAGAAGAG  ATATGCCCATTTAACCACGC | 676 | 60 | Hong et al., 2018 |
|  | *tet*(W) | TETW-F  TETW-R | GAGAGCCTGCTATATGCCAGC  GGGCGTATCCACAATGTTAAC | 168 | 50 | Jiang et al., 2013 |
|  | *tet*(K) | *tet*(X)-F  *tet*(X)-R | TCGATAGGAACAGCAGTA  CAGCAGATCCTACTCCTT | 169 | 61 | Hong et al., 2018 |
| **Erythromycin** | *erm*B | ERMB -F  ERMB -R | GCATTTAACGACGAAACTGGCT  GACAATACTTGCTCATAAGTAATGGT | 573 | 61 | Hong et al., 2018 |
| **Colistin** | *mcr-*1 | mcr-1-F  mcr-1-R | TATCGCTATGTGCTAAAGCCTG  CGTCTGCAGCCACTGGG | 1139 | 56 | Jousset et al., 2019 |
|  | *mcr-*2 | mcr-2-F  mcr-2-R | TATCGCTATGTGCTAAAGCCTG  AAAATACTGCGTGGCAGGTAGC | 816 | 56 | Jousset et al., 2019 |
|  | *mcr-*3 | mcr-3-F  mcr-3-R | CAATCGTTAGTTACACAATGATGAAG  AACACATCTAGCAGGCCCTC | 676 | 56 | Jousset et al., 2019 |
|  | *mcr-*4 | mcr-4-F  mcr-4-R | ATCCTGCTGAAGCATTGATG  GCGCGCAGTTTCACC | 405 | 56 | Jousset et al., 2019 |
|  | *mcr-*5 | mcr-5-F  mcr-5-R | GGTTGAGCGGCTATGAAC  GAATGTTGACGTCACTACGG | 207 | 56 | Jousset et al., 2019 |
| **Chloramphenicol** |  |  |  |  |  |  |
|  | *cat*I | catI-F  catI-R | GGTGATATGGGATAGTGTT  CCATCACATACTGCATGATG | 349 | 60 | Hong et al., 2018 |
|  | *cat*II | catII-F  catII-R | GATTGACCTGAATACCTGGAA  CCATCACATACTGCATGATG | 567 | 60 | Hong et al., 2018 |
|  | *cat*III | catIII-F  CatIII-R | CCATACTCATCCGATATTGA  CCATCACATACTGCATGATG | 275 | 60 | Jiang et al., 2013 |
|  | *cat*IV | CatIV-F  catIV R | CCGGTAAAGCGAAATTGTAT  CCATCACATACTGCATGATG | 451 | 60 | Hong et al., 2018 |
|  | *flo*R | FloR-F  FloR-R | CGCCGTCATTCCTCACCTTC  GATCACGGGCCACGCTGTGTC | 215 | 50 | Jiang et al., 2013 |
| **Sulfonamide** |  |  |  |  |  |  |
|  | *sul*I | sulI-F  sulI-R | CGCACCGGAAACATCGCTGCAC  TGAAGTTCCGCCGCAAGGCTCG | 163 | 63 | Hong et al., 2018 |
|  | *sul*II | sulII-F  sulII-R | TCCGGTGGAGGCCGGTATCTGG  CGGGAATGCCATCTGCCTTGAG | 191 | 63 | Hong et al., 2018 |
|  | *sul*III | sulIII-F  sulIII-R | TCCGTTCAGCGAATTGGTGCAG  TTCGTTCACGCCTTACACCAGC | 128 | 61 | Hong et al., 2018 |
| **β-lactam** |  |  |  |  |  |  |
|  | *amp*C | AmpC -F  AmpC R | GTGACCAGATACTGGCCACA  TTACTGTAGCGCCTCGAGGA | 822 |  | Liu et a., 2018 |
|  | *SHV* | SHV-F  SHV-R | CACTCAAGGATGTATTGT G  TTAGCGTTGCCAGTGCTCG | 885 | 55 | Ramatla et al., 2022 |
|  | *OXA* | OXA-F  OXA -R | ACACAATACATATCAACTTCGC  AGTGTGTTTAGAATGGTGATC | 813 | 55 | Ramatla et al., 2022 |
|  | *CARB* | CARB-F  CARB-R | CAAGTACTTTYAAAACAATAGC  GCTGTAATACTCCKAGCAC | 534 | 46 | Jiang et al., 2013 |
|  | *TEM* | TEM-F  TEM-R | TTC TTG AAG ACG AAA GGG C  ACGCTCAGTGGAACGAAAAC | 1150 | 55 | Ramatla et al., 2022 |
|  | *CTX-M* | CTX-M-F  CTX-M-R | GTTACAATGTGTGAGAAGCAG  CCGTTTCCGCTATTACAAAC | 550 | 55 | Liu et al., 2018 |
|  | *CTX-M-1* group | CTX-M-1-F  CTX-M-1-R | GTT ACA ATG TGT GAG AAG CAG  CCGTTTCCGCTATTACAAAC | 1041 | 55 | Liu et al., 2018 |
|  | *CTX-M-2* group | CTX-M-2-F  CTX-M-2-R | ATGATGACTCAGAGCATTCGCCGC  TCAGAAACCGTGGGTTACGATTTT | 876 | 56 | Gundran et al., 2019 |
|  | *CTX-M-8* group | CTX-M-8-F  CTX-M-8-R | TGATGAGACATCGCGTTAAG  TAACCGTCGGTGACGATTTT | 666 | 52 | Gundran et al., 2019 |
|  | *CTX-M-9* group | CTX-M-9-F  CTX-M-9-R | GTGACAAAGAGAGTGCAACGG  ATGATTCTCGCCGCTGAAGCC | 856 | 55 | Gundran et al., 2019 |
|  | *CTX-M-15* group | CTX-M-15  CTX-M-15-R | CACACGTGGAATTTAGGGACT  GCCGTCTAAGGCGATAAACA | 995 | 50 | Gundran et al., 2019 |
|  | *CTX-M-25* group | CTX-M-25-F  CTX-M-25-R | GCACGATGACATTCGGG  AACCCACGATGTGGGTAGC | 327 | 52 | Gundran et al., 2019 |
| **Quinolone** |  |  |  |  |  |  |
|  | *qnr*A | qnrA-F  qnrA-R | ATTTCTCACGCCAGGATTTG  GAGATTGGCATTGCTCCAGT | 413 | 56 | Xu et al., 2015 |
|  | *qnr*D | qnrD-F  qnrD-R | GCTGGAGCTTGTCAGGGATT  TGCTGCGAGATATCATGCGT | 585 | 59 | Xu et al., 2015 |
|  | *qnrS* | qnrS-F  qnrS-R | CCCCATGCCCGAAGTTATCA  ACTGCTTGGAGTGTGTTGGT | 457 | 53 | Sun et al., 2016 |
|  | *par*C | parC-F  parC-R | GCCTAAACAACGCACGGAAA  TGACACGGGAGGTAACCAGA | 432 | 59 | Xu et al., 2015 |
| **Aminoglycoside** |  |  |  |  |  |  |
|  | *str*A | strA-F  strA-R | CTTGGTGATAACGGCAATTC  CCAATCGCAGATAGAAGGC | 548 | 55 | Hong et al., 2018 |
|  | *str*B | strB-F  strB-R | ATCGTCAAGGGATTGAAACC  GGATCGTAGAACATATTGGC | 509 | 56 | Hong et al., 2018 |
|  | *aad*A | aadA-F  aadA-R | ATCCTTCGGCGCGATTTTG  GCAGCGCAATGACATTCTTG | 283 | 56 | Hong et al., 2018 |
|  |  |  |  |  |  |  |

**Reference**

Sun W, Qian X, Gu, J, Wang XJ, Duan, ML (2016) Mechanism and effect of temperature on variations in antibiotic resistance genes during anaerobic digestion of dairy manure. *Scientific reports*, *6*(1), p.30237.

Ouoba L, Lei V, Jensen LB (2008) Resistance of potential probiotic lactic acid bacteria and bifidobacteria of African and European origin to antimicrobials: Determination and transferability of the resistance genes to other bacteria. Int. J. Food Microbiol 121(2), 217–224.

Jiang L, Hu XL, Xu T, Zhang HC, Sheng D, Yin DQ (2013) Prevalence of antibiotic resistance genes and their relationship with antibiotic in the Huangpu River and the drinking water sources, Shanghai, China. Sci. Total Environ 458-460, 267-272.

Hong B, Ba Y, Niu L, Lou F, Zhang Z, Liu H, Pan Y, Zhao Y (2018) A comprehensive research on antibiotic resistance genes in microbiota of aquatic animals. Front Microbiol 9: 1617.

Gundran RS, Cardenio PA, Villanueva MA, Sison FB, Benigno CC, Kreausukon K, Pichpol D, Punyapornwithaya V (2019) Prevalence and distribution of bla CTX-M, bla SHV, bla TEM genes in extended-spectrum β-lactamase-producing E. coli isolates from broiler farms in the Philippines. BMC Vet. Res 15:1-8.

Liu G, Ding L, Han B, Piepers S, Naqvi SA, Barkema HW, Ali T, De Vliegher S, Xu S, Gao J (2018) Characteristics of Escherichia coli isolated from bovine mastitis exposed to subminimum inhibitory concentrations of cefalotin or ceftazidime. Biomed Res. Int 2018;2018.

Liu G, Ding L, Han B, Piepers S, Naqvi SA, Barkema HW, Ali T, De Vliegher S, Xu S, Gao J (2018) Characteristics of Escherichia coli isolated from bovine mastitis exposed to subminimum inhibitory concentrations of cefalotin or ceftazidime. Biomed Res. Int 2018: 4301628

Ramatla T, Mileng K, Ndou R, Mphuti N, Syakalima M, Lekota KE, Thekisoe OM (2022) Molecular detection of integrons, colistin and β-lactamase resistant genes in Salmonella enterica serovars enteritidis and typhimurium isolated from chickens and rats inhabiting poultry farms. Microorganisms 10(2):313.
